# Targeting farnesylation as a novel therapeutic approach in HRAS-mutant rhabdomyosarcoma

**DOI:** 10.1038/s41388-022-02305-x

**Published:** 2022-04-22

**Authors:** Patience Odeniyide, Marielle E. Yohe, Kai Pollard, Angelina V. Vaseva, Ana Calizo, Lindy Zhang, Fausto J. Rodriguez, John M. Gross, Amy N. Allen, Xiaolin Wan, Romel Somwar, Karisa C. Schreck, Linda Kessler, Jiawan Wang, Christine A. Pratilas

**Affiliations:** 1 Division of Pediatric Oncology, The Sidney Kimmel Comprehensive Cancer Center, Johns Hopkins University School of Medicine, Baltimore, MD USA; 2 Pediatric Oncology Branch, National Cancer Institute, National Institutes of Health, Bethesda, MD 20892 USA; 3 The Greehey Children’s Cancer Research Institute, The University of Texas Health Science Center at San Antonio, San Antonio, TX USA; 4 Department of Laboratory Medicine and Pathology, David Geffen School of Medicine, UCLA, Los Angeles, CA USA; 5 Department of Pathology, Johns Hopkins University School of Medicine, Baltimore, MD USA; 6 Department of Pathology, Memorial Sloan Kettering Cancer Center, New York, NY USA; 7 Department of Oncology, The Sidney Kimmel Comprehensive Cancer Center, Johns Hopkins University School of Medicine, Baltimore, MD USA; 8 Department of Neurology, Johns Hopkins University School of Medicine, Baltimore, MD USA; 9 Kura Oncology, San Diego, CA USA

**Keywords:** Cancer genomics, Sarcoma

## Abstract

Activating RAS mutations are found in a subset of fusion-negative rhabdomyosarcoma (RMS), and therapeutic strategies to directly target RAS in these tumors have been investigated, without clinical success to date. A potential strategy to inhibit oncogenic RAS activity is the disruption of RAS prenylation, an obligate step for RAS membrane localization and effector pathway signaling, through inhibition of farnesyltransferase (FTase). Of the major RAS family members, HRAS is uniquely dependent on FTase for prenylation, whereas NRAS and KRAS can utilize geranylgeranyl transferase as a bypass prenylation mechanism. Tumors driven by oncogenic HRAS may therefore be uniquely sensitive to FTase inhibition. To investigate the mutation-specific effects of FTase inhibition in RMS we utilized tipifarnib, a potent and selective FTase inhibitor, in in vitro and in vivo models of RMS genomically characterized for RAS mutation status. Tipifarnib reduced HRAS processing, and plasma membrane localization leading to decreased GTP-bound HRAS and decreased signaling through RAS effector pathways. In HRAS-mutant cell lines, tipifarnib reduced two-dimensional and three-dimensional cell growth, and in vivo treatment with tipifarnib resulted in tumor growth inhibition exclusively in HRAS-mutant RMS xenografts. Our data suggest that small molecule inhibition of FTase is active in HRAS-driven RMS and may represent an effective therapeutic strategy for a genomically-defined subset of patients with RMS.

## Introduction

Aberrant RAS activation is implicated as a major driver in up to 30% of human cancers [1–3]. Efforts to inhibit mutant RAS directly, however, have historically met with failure [4–6] leading to the notion that RAS is “undruggable”. Various strategies have been employed to inhibit oncogenic RAS [7], including disruption of signaling pathways both upstream and downstream of RAS [6], direct RAS inhibition through design of mutant allele-specific compounds [8], inhibition of RAS recruitment and activating proteins, such as SHP2 and SOS [9–11], and the inhibition of RAS membrane localization. RAS proteins require prenylation, the addition of a lipid moiety to the CAAX motif, which promotes association with the lipid bilayer of the plasma membrane [12]. This obligate post-translational modification, via the enzymes farnesyltransferase (FTase) and geranyl-geranyl transferase (GGTase), facilitates membrane localization and signal transduction through RAS effector pathways [13]. GGTase-mediated prenylation represents an alternate or “bypass” prenylation mechanism utilized by NRAS and KRAS molecules to escape from FTase inhibition [14]. Alternate prenylation by GGTase has been cited as a major limitation in the ability of FTase inhibitors (FTI) to effectively inhibit RAS-driven cancers [15]. HRAS, however, is exclusively dependent on FTase for prenylation and therefore FTase inhibition may be a therapeutic strategy in tumors in which HRAS is a driver oncogene.

Tipifarnib (Kura Oncology) is a potent and highly selective non-peptidomimetic competitive inhibitor of the CAAX peptide binding site of FTase [16]. The inhibitory effects of tipifarnib have been reported in various in vitro models including acute myeloid leukemia [17], lymphoma [18], and triple-negative breast cancer [19]. In the early 2000’s a number of clinical trials were conducted with tipifarnib, both in adult [20–25] and in pediatric [26–28] patients. Other than a few modest responses in hematologic malignancies [29], however, each of these trials failed to demonstrate sufficient activity to support its advancement to later-stage clinical trials [25, 30, 31]. Recently, however, there have been rekindled efforts to exploit the reliance of HRAS on FTase. A study of tipifarnib in HRAS-mutant specific models demonstrated that tipifarnib inhibits tumor growth in patient-derived xenograft (PDX) models of head and neck squamous cell carcinoma (HNSCC) [32] and transgenic murine models of thyroid cancer [33]. Early phase trials have demonstrated clinical responses to tipifarnib in patients with HRAS-mutated squamous cell carcinomas (SCC) [34], recurrent and metastatic salivary gland carcinomas [35], and advanced and refractory urothelial carcinomas [36]. No pediatric clinical trial completed to date, however, has evaluated the efficacy of FTI specifically in patients whose tumors harbor activating mutations in HRAS [37, 38].

RAS mutations (including those in *HRAS, NRAS* and *KRAS*) occur in approximately 25% of cases of fusion-negative (embryonal) rhabdomyosarcoma (RMS) and may occur at a higher frequency in younger patients [39, 40]. Our goal, therefore, was to determine whether FTase inhibition using tipifarnib could be a viable therapeutic strategy for these RMS patients and those with other HRAS-driven solid malignancies. We hypothesized that when tested in RMS cell lines, xenografts or PDX that harbor activating mutations in HRAS, FTase inhibition will elicit genotype-dependent anti-tumor activity. Data in support of this hypothesis could then be used to justify a histology-agnostic basket trial of tipifarnib in pediatric patients with HRAS-driven solid tumors, such as the ongoing trial being conducted under the Children’s Oncology Group Pediatric MATCH (Molecular Analysis for Therapy Choice) program (NCT03155620).

## Results

### Tipifarnib disrupts HRAS processing and plasma membrane localization in RMS cell lines

**Fig. 1:**
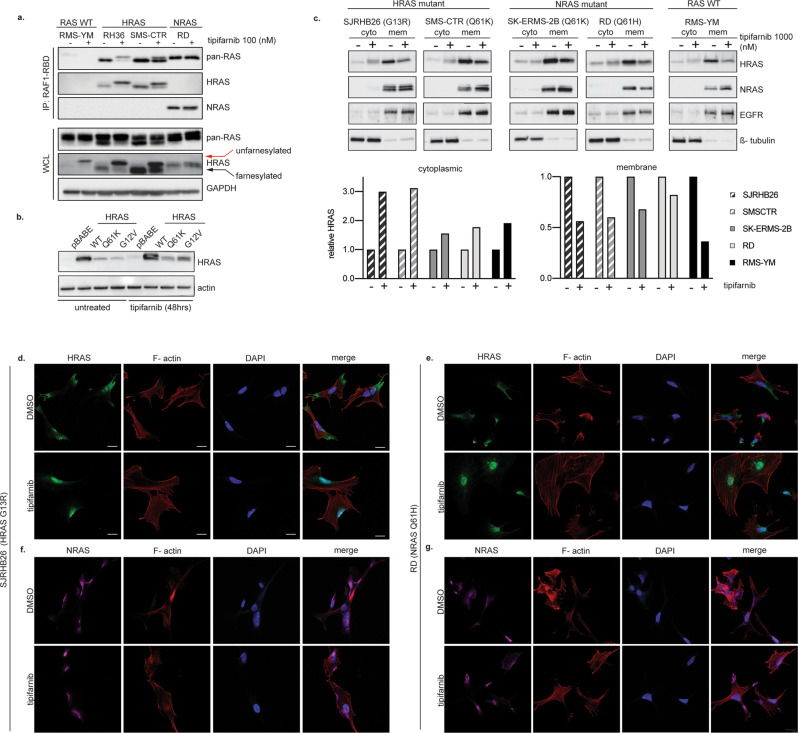
Tipifarnib decreases HRAS processing and plasma membrane localization. **a** RMS cell lines were treated with 100 nM tipifarnib or DMSO for 24 h and subjected to immunoprecipitation and immunoblot. **b** Wild-type HRAS, HRAS_Q61K and HRAS_G12V mutants in C2C12 cells were treated with 100 nM tipifarnib for 48 h. HRAS and actin (loading control) were determined by immunoblot from whole cell lysate (WCL). **c** Cytosolic and membrane fractions of RMS cell lines treated with 1000 nM tipifarnib or DMSO for 24 h. The intensity of HRAS was determined by densitometric analysis using Image J. SJRHB000026_X1 (HRAS G13R, abbreviated SJRHB26 throughout figures) and RD (NRAS Q61H) were treated with either DMSO or 1000 nM tipifarnib for 24 h and were subjected to immunofluorescent staining for HRAS (green) or NRAS (magenta) along with filamentous actin (F-actin) (red) and 4’,6-diamidino-2-phenylindole (DAPI) nuclear stain (blue). The localization of HRAS was analyzed by con-focal immunofluorescence microscopy. Pictures show areas of similar cell density. Scale bar = 20 μM. Cellular distribution of HRAS in response to tipifarnib (**d**, **e**). Distribution of NRAS in response to tipifarnib (**f**, **g**).

Using a genomically characterized panel of RMS cell lines with known mutations in *HRAS, NRAS, KRAS* and a subset of other genes (Table 1), we first sought to determine the effects of tipifarnib on HRAS farnesylation. Following treatment with tipifarnib, we used affinity purification with the RAS binding domain of RAF1 (RAF1-RBD), in order to isolate the GTP-bound fraction of RAS in the cells. The predominant GTP-bound RAS in HRAS-mutant cells was HRAS-GTP, as anticipated. NRAS was uniquely GTP-bound in NRAS mutant cells, and in RAS wild-type (WT) cells, low levels of RAS-GTP were detected (Fig. 1a). Tipifarnib led to a mobility shift, and in some cases the emergence of a second band, on immunoblot for HRAS, but not NRAS (Fig. 1a, red arrow). This effect, representing unfarnesylated HRAS, was recapitulated in isogenic cells transfected with various mutant HRAS forms (Fig. 1b), and is the result of slower mobility of non-prenylated proteins through SDS-PAGE gels [8, 41]. Similar changes in RAC and RhoA were not observed in our experiments (Supplemental Fig. 2), consistent with reports of their ability to undergo geranylgeranylation [42, 43].

**Table 1:**
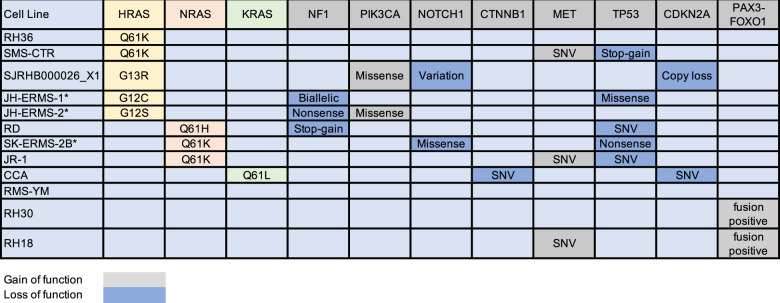
Genomic characterization of RMS cell lines used in the current study.

*Novel JHU or MSKCC patient-derived cell lines.

Yellow color designates HRAS-mutant cell lines. Orange designates NRAS-mutant cell lines and green designates KRAS-mutant cell lines.

We next examined the effects of tipifarnib on RAS membrane localization, as farnesylation is required for membrane localization and therefore RAS activation. Using subcellular fractionation to isolate cytoplasmic- and membrane-bound protein fractions, we found that tipifarnib increased HRAS in the cytosolic fraction and decreased HRAS in the membrane fraction, compared to untreated cells, independently of RAS mutation status. The quantity of NRAS in the membrane and cytosolic fractions, using the same method, was not affected by treatment with tipifarnib (Fig. 1c). We further examined membrane localization using immunofluorescence, and found that tipifarnib reduced HRAS membrane localization, irrespective of RAS mutation status, and resulted in cytoplasmic pooling, and in fact, some nuclear localization of HRAS as well, in treated cell lines compared to control (Fig. 1d, e). As expected, tipifarnib did not alter or reduce NRAS membrane localization (Fig. 1f, g), again, irrespective of cell genotype. This observation is consistent with activation of the alternate pathway for prenylation and plasma membrane localization utilized by NRAS and KRAS [44].

### ERK signaling is attenuated by tipifarnib in HRAS-mutant cell lines

**Fig. 2:**
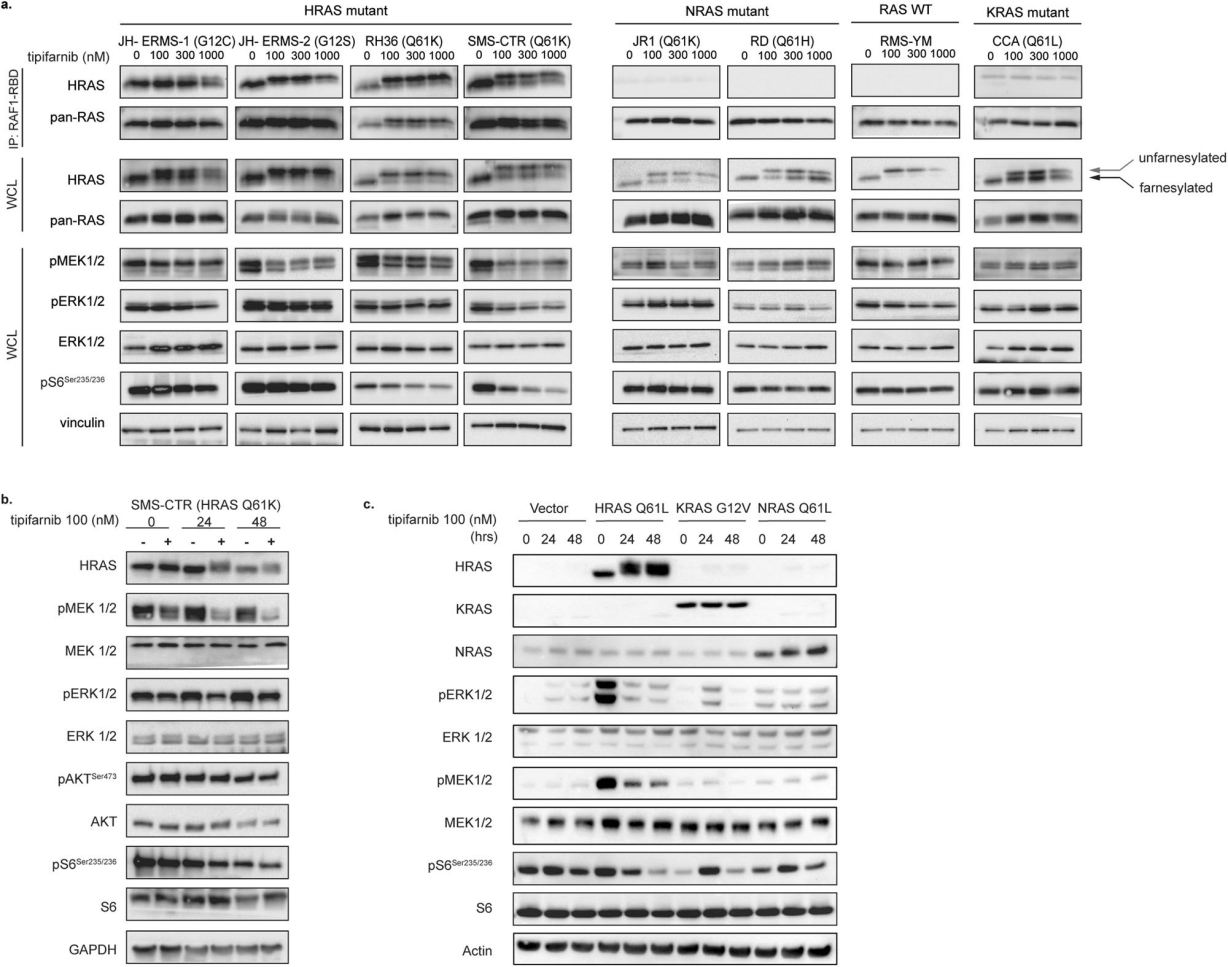
Tipifarnib inhibits HRAS farnesylation and ERK signaling. **a** A panel of RMS cell lines (mutations as indicated) were treated with DMSO, 100, 300, 1000 nM tipifarnib for 24 h. Activated RAS protein (RAS-GTP) was quantitated via immunoprecipitation with the RAS-binding domain of RAF (RAF1-RBD), followed by immunoblot using pan-RAS and HRAS antibody. Arrows represent the accumulation of unfarnesylated HRAS (gray arrow) in response to tipifarnib in comparison to farnesylated HRAS (black arrow). Phospho- and total levels of ERK pathway components, and vinculin (loading control) were determined by immunoblot from whole cell lysate (WCL). **b** SMS-CTR was treated with DMSO or 100 nM tipifarnib for 0, 24, 48 h. Phospho- and total levels of ERK pathway components, and GAPDH (loading control) were determined by immunoblot from whole cell lysate (WCL). **c** HRAS_Q61L, KRAS_G12V, NRAS_Q61L mutants, or vector alone, were expressed in C2C12 cells and then treated with 100 nM tipifarnib for 0, 24 or 48 h. Phospho- and total levels of ERK and PI3K pathway components, and actin (loading control) were determined by immunoblot from whole cell lysate (WCL).

We hypothesized that inhibition of HRAS membrane localization via tipifarnib would decrease signaling through RAS effector pathways including MEK/ERK and PI3K/mTOR. To evaluate this effect, we incubated RMS cell lines (including two novel patient-derived cell lines generated in our lab, JH-ERMS-1 and JH-ERMS-2, see Methods for details on development and characterization) with varying concentrations of tipifarnib and measured RAS activation and downstream signaling effects. Detection of GTP-bound RAS revealed that HRAS-mutant cell lines exhibited high levels of active, GTP-bound HRAS compared with HRAS WT cell lines (those with NRAS mutation, those with KRAS mutation, and those with WT RAS) which exhibited undetectable levels of HRAS-GTP (Fig. 2a).

We next assessed the effects of tipifarnib on phosphorylation of MEK1/2 and ERK1/2, and phospho-S6 as a readout of PI3K/mTOR pathway activity. Treatment of HRAS-mutant cells with tipifarnib resulted in decreased phosphorylation of MEK1/2 (pMEK) and S6 (pS6^Ser235/236^) at 24 h in most HRAS-mutant cell lines, although more potently in some lines compared to others. The degree of ERK phosphorylation inhibition was more varied, and was most potently downregulated by tipifarnib in the lines JH-ERMS-1 and SMS-CTR (Fig. 2a, b). NRAS and KRAS-mutant cell lines, and WT RAS cell lines, demonstrated no detectable changes in MEK1/2, ERK1/2, or S6 phosphorylation in response to tipifarnib (Fig. 2a). The effects of tipifarnib were mostly cytostatic, although modest induction of PARP and caspase cleavage were noted in one cell line, SMS-CTR, which was consistently the most sensitive HRAS-mutant cell line tested (Supplemental Fig. 3a). Markers of apoptosis were absent in the remainder of cell lines tested (Supplemental Fig. 3c).

To validate these effects of tipifarnib on ERK signaling in an isogenic model system, we utilized C2C12 murine myoblast cells stably expressing KRAS G12V, HRAS Q61L and NRAS Q61L, or vector alone as control [45]. Similar to our observations in established RMS cell lines, we found that in cells transduced with HRAS Q61L, but not in those transduced with mutant NRAS or KRAS, signaling downstream of RAS (including pMEK, pERK, and pS6) was inhibited (Fig. 2c). We therefore concluded that the effects of FTase inhibition with tipifarnib on the MEK/ERK and PI3K/mTOR pathways are selectively dependent upon the HRAS-mutant genotype.

### Tipifarnib selectively decreases anchorage-dependent and -independent cell growth in HRAS-mutated RMS cell lines

**Fig. 3:**
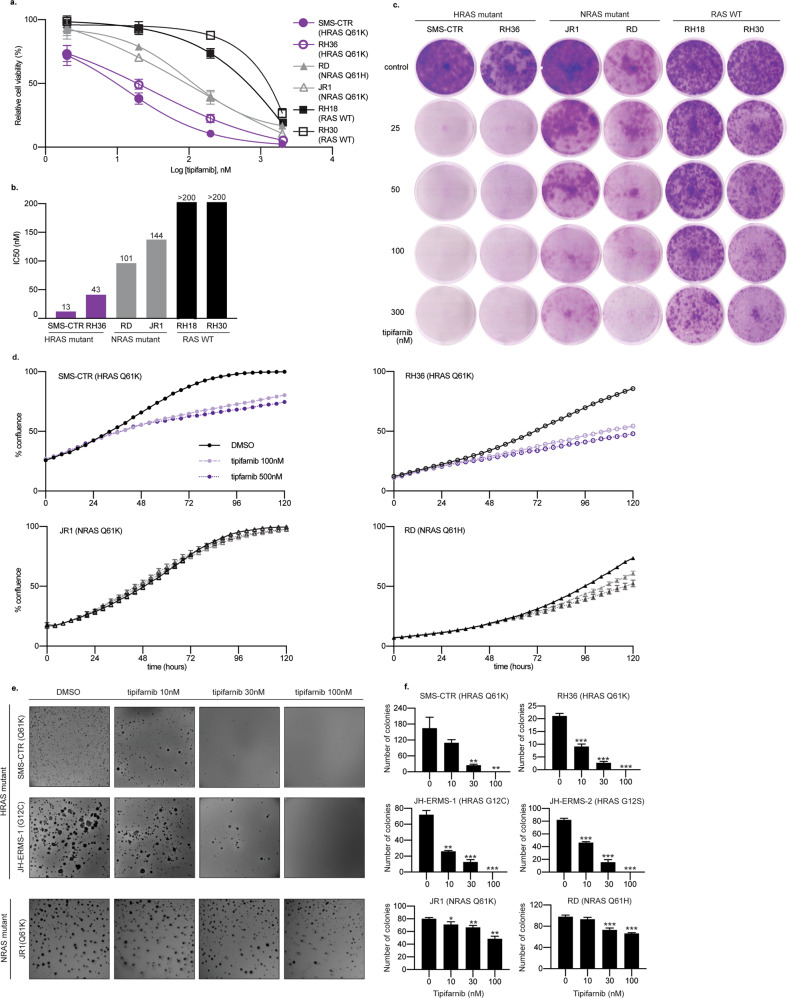
Tipifarnib decreases anchorage-dependent and -independent growth in HRAS-mutated cell lines. **a** Six RMS cell lines were treated with DMSO or increasing doses of tipifarnib for 96 h. Percent viability is shown normalized to DMSO control. **b** Half maximal inhibitory concentration (IC_50_) values were determined using a four-parameter fit nonlinear regression analysis. Error bars represent mean of three measurements ± SD of mean. **c** Six RMS cell lines were treated with DMSO or indicated doses of tipifarnib for two weeks. Cells were fixed and stained with 0.1% crystal violet in 4% paraformaldehyde for 20 min. Representative images are shown. **d** Cell confluency (%) was calculated using IncuCyte ZOOM software based on phase-contrast images of SMS-CTR, RH36, RD, JR1 cells from 0 to 120 h at 0, 100 nM and 500 nM tipifarnib. Each data point represents six wells. **e** Representative images of RMS cell lines grown in soft agar and treated with either DMSO, 10, 30 or 100 nM tipifarnib for three weeks. **f** The y-axis represents the absolute number of colonies, three wells per condition, for each cell line. Error bars represent standard error (SEM) of three technical replicates. *p < 0.05, **p < 0.01, ***p < 0.001, unpaired Student *t*-test. Statistical comparisons are relative to control.

We next sought to determine the genotype-dependent effects of tipifarnib on in vitro growth and proliferation. We exposed RMS cell lines to increasing doses of tipifarnib (in a range from 0 to 2000 nM) using high-throughput cell proliferation, real-time confluence monitoring, and colony-forming assays. HRAS-mutant cell lines demonstrated marked sensitivity, and lower IC_50_s to tipifarnib in comparison to NRAS-mutant and WT RAS (fusion-positive) cell lines (Fig. 3a–c) using a standard metabolism-based cell viability assay (alamarBlue). In our experiments, SMS-CTR (HRAS Q61K) was the most sensitive cell line to tipifarnib, with an IC_50_ of 13 nM (Fig. 3b). Using the IncuCyte ZOOM live-cell imaging system, we monitored growth of established cell lines in the presence of tipifarnib, and observed only modest growth inhibitory effects, again limited to the HRAS-mutant cell lines (Fig. 3d). As this assay measures confluence rather than direct assessment of cell proliferation, it is quite possible that as cells differentiate and therefore enlarge, this assay underestimates the overall effect of tipifarnib on cells growing in 2D. We therefore utilized soft agar colony formation assays as in vitro representation of anchorage-independent growth and tumorigenicity. Tipifarnib consistently had a marked effect on the colony-forming capacity on all HRAS-mutant cell lines and suppressed colony formation completely at the highest dose evaluated (100 nM) (Fig. 3e, f). Cells with NRAS mutations exhibited little sensitivity and formed numerous colonies at the highest dose evaluated (Fig. 3e, f). These data support our hypothesis that FTase inhibition with tipifarnib decreases HRAS membrane localization and signaling through MEK/ERK and PI3K/mTOR pathways, thereby inhibiting growth of only cells with oncogenic HRAS.

### Tipifarnib selectively suppresses growth of HRAS-mutated xenograft tumors

**Fig. 4:**
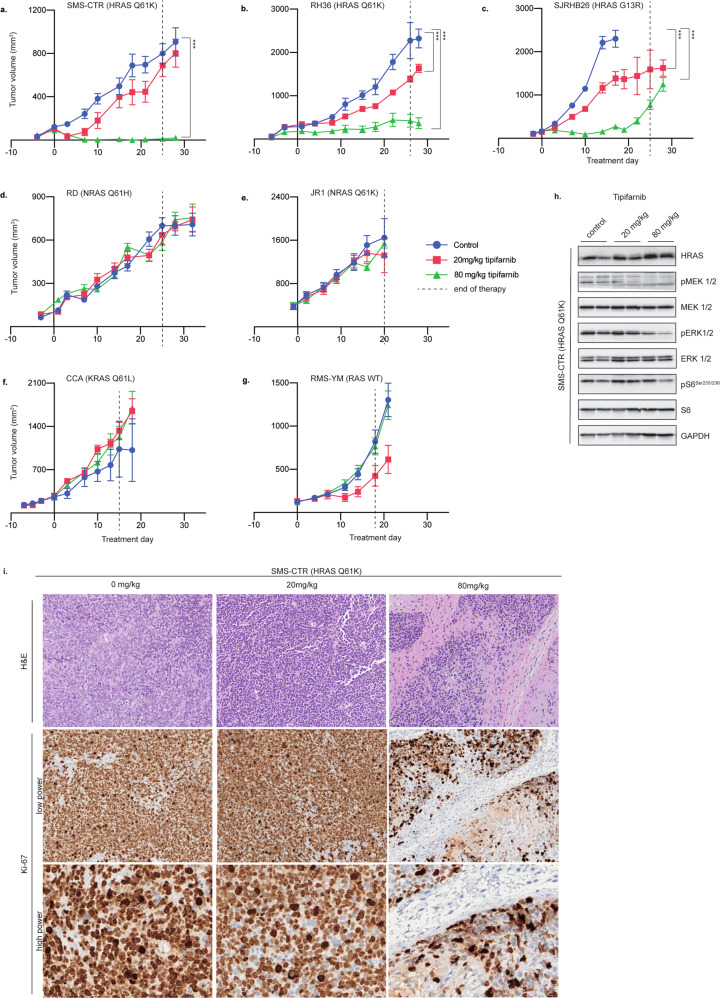
Tipifarnib preferentially inhibits growth of HRAS-mutated RMS xenografts. NSG mice bearing HRAS-mutant (**a**–**c**), NRAS-mutant (**d**, **e**), KRAS-mutant (**f**), and RAS WT (**g**) xenografts were treated with vehicle, or tipifarnib at 20 or 80 mg/kg twice daily (5 days on/2 days off) for three weeks. Tumor volumes were calculated twice weekly. The average tumor volume is graphed as a function of days on treatment. Error bars represent mean ± SEM. *p < 0.05, **p < 0.01, ***p < 0.001, unpaired Student *t*-test. Statistical comparisons are relative to control groups on treatment day 28. **h** NSG mice bearing SMS-CTR (HRAS Q61K) xenografts were treated with vehicle, or tipifarnib at 20 or 80 mg/kg twice daily for 10 doses. Phospho- and total levels of ERK and PI3K pathway components, and GAPDH (loading control) were determined by immunoblot from whole cell lysate (WCL). **i** NSG mice bearing SMS-CTR (HRAS Q61K) xenografts were treated with vehicle, or tipifarnib at 20 or 80 mg/kg twice daily for (5 days on/2 days off) for three weeks. Tumor extracts were stained using hematoxylin and eosin (H&E) and subject to immunohistochemistry and stained for Ki-67.

To validate the efficacy of tipifarnib in inhibiting growth of HRAS-mutant tumors, we examined its efficacy in heterotopically implanted subcutaneous murine xenograft models. The HRAS-mutant xenografts as a group were significantly more sensitive to tipifarnib than NRAS-mutant, KRAS-mutant, and WT RAS xenografts. At 80 mg/kg twice daily, tipifarnib markedly suppressed the growth of HRAS xenografts compared to vehicle controls (Fig. 4a–c). The effects were primarily cytostatic, with only minimal induction of cleaved PARP in one xenograft model (Supplemental Fig. 3b). In each of the HRAS-mutant models, tumors regrowth occurred after cessation of exposure to tipifarnib, with mild tumor regression on retreatment (Supplemental Fig. 4). In contrast, murine xenografts with NRAS or KRAS mutations or WT RAS were insensitive to tipifarnib at all dose levels (Fig. 4d–g). In all cohorts, no adverse effects of drug treatment on body weight were observed. These data provide further evidence that the genotype-selective effects of tipifarnib are limited to those with HRAS mutations, but not specific to the codon alterations (i.e., Q61 versus G13 variants). Tumor extracts from a cohort of SMS-CTR xenografts (HRAS Q61K) demonstrated a decrease in downstream effectors (pMEK, pERK, pS6) via immunoblot, further demonstrating the efficacy of tipifarnib in this cohort (Fig. 4h). Subsequent application of Ki-67 immunohistochemistry in SMS-CTR xenograft showed a dose-dependent decrease in nuclear expression suggesting a possible decrease in tumor proliferation in response to tipifarnib (Fig. 4i and Supplemental Fig. 5). Moreover, careful histologic review demonstrates cytologic evidence of tumor maturation as scattered cells are seen producing abundant rhabdoid cytoplasm in the tipifarnib 80 mg/kg cohort.

## Discussion

Efforts in drug development to selectively target RAS have been a major research and discovery focus since the recognition of RAS as oncoproteins in 1982 [46, 47] and subsequent studies that demonstrate that RAS is a driver oncogene in up to 24% of all human cancers [4]. Despite extensive scientific research, clinical trials, and large-scale government commitments to this purpose, only one drug that directly targets mutant RAS has received regulatory approval to date, and its activity is limited to tumors with the specific KRAS mutation G12C [48, 49]. Among the proposed approaches to RAS signaling inhibition in cancer, one concept that is clinically compelling is disruption of RAS plasma membrane localization [50] and thereby interruption of RAS guanine nucleotide exchange and signaling to RAS effector pathways [51]. The post-translational modification of RAS that is required for membrane association involves prenylation catalyzed by FTase (HRAS, NRAS and KRAS) and GGTase (NRAS and KRAS only) [44]. Given the exclusive reliance of HRAS proteins on FTase for membrane localization and activity, FTase inhibition has emerged as an attractive therapeutic strategy for HRAS-driven cancers.

FTase inhibitors were developed in the 1990s, but demonstrated limited efficacy in clinical trials involving patients with multiple tumor types [25, 31, 41, 52]. Given the early stage and limited availability of clinical tumor sequencing at that time, however, patient enrollment and selection were not restricted based on genotype, a concept which has now emerged as critical in the trial design of many molecularly targeted agents, the activity of which may be genotype-selective [53, 54]. Enrollment of patients with tumors bearing other oncogenic forms of RAS, is at least in part responsible for the modest clinical responses seen at that time. Recent and emerging clinical data using tipifarnib in patients with HRAS-mutant HNSCC [55], salivary gland cancer [35], and metastatic urothelial carcinoma [36, 56] suggest that higher rates of clinical activity will be realized in a molecularly-defined population, which led to renewed interest in examining the efficacy of tipifarnib in tumors with hotspot *HRAS* mutations.

RAS mutations are seen in up to one-third of fusion-negative (absence of PAX3- or PAX7-FOXO1 fusion) RMS and may be disproportionately seen in younger patients with RMS [39]. Attempts to augment cytotoxic chemotherapy regimens have not improved outcomes in various subsets of RMS patients [57, 58], and high-risk RMS represents a major unmet need in pediatric oncology. We therefore set out to determine whether FTase inhibition with tipifarnib is a viable therapeutic approach for patients with HRAS-mutated RMS.

To evaluate the efficacy of tipifarnib in RMS we collected a large panel of RMS cell lines including those with *HRAS*, *NRAS*, *KRAS* mutations and RAS WT (including fusion-negative and fusion-positive subtypes). We included cell lines with a range of mutant alleles (Q61K, Q61L, Q61H, G12C, G12S, and G13R) to allow us to study the in vitro and in vivo effects of FTase inhibition in the setting of diverse concurrent mutations and to identify any potential allele-specific effects. We demonstrated that tipifarnib inhibits prenylation of mutant and WT HRAS leading to decreased plasma membrane loading, consistent with prior studies [32, 33]. Inhibition of HRAS membrane localization via FTIs has been shown to lead to a cytosolic accumulation of unfarnesylated HRAS that can bind to but not activate CRAF [8, 59]. Of note, our results also demonstrated some localization of HRAS to the nucleus, consistent with published studies that demonstrated cyclic movement of HRAS between cytosolic and nuclear compartments in both non-transformed and RAS-transformed cells [60, 61]. Our data suggest modulation of HRAS prenylation and GTP binding by tipifarnib, and consequent reduction of ERK signaling downstream of CRAF, as previously described [32, 33, 56]. Importantly, inactivation of the PI3K pathway intermediate pS6^Ser235/236^ was also seen in HRAS-mutant cell lines, indicating concomitant inhibition of potential survival pathways. Modest pERK inhibition was seen in some HRAS-mutant cell lines despite more significant pMEK inhibition. This finding has been seen in additional HRAS-mutant models [33] and warrants further exploration. Despite the seemingly insufficient inhibition of pERK, we observed a robust inhibition of anchorage-independent growth and tumor growth in vivo, suggesting that other farnesylated proteins (including Rheb, Rac1, RhoB, mTOR/Raptor, lamins A/B and CENP-E/CENP-F [62–64]) outside of the MEK/ERK pathway could be contributing to the growth inhibitory effects of tipifarnib.

In our models of RMS, tipifarnib selectively inhibited tumor growth in HRAS-mutant xenografts early in the treatment course and without toxicity, consistent with other mouse models of HRAS-mutant cancers [32, 33, 56]. Growth inhibition was most sustained in the SMS-CTR xenograft (HRAS Q61K) which was our most sensitive cell line in in vitro studies as well. HRAS-mutant models tumors regrew after cessation of exposure to tipifarnib, with mild tumor regression on retreatment (Supplemental Fig. 4), indicating that FTase inhibition may confer only a cytostatic effect on tumor growth. Cellular morphology also revealed hints of a differentiation response, and although preliminary, these findings together may suggest that treatment will be optimized using alternate dose schedules and/or combination strategies.

It is likely that among tumors, and therefore patients, with *HRAS* mutations, variable responses will be observed, due to other genomic alterations, both pre-existing and treatment-emergent. It will therefore be prudent to investigate the contribution of co-occurring genomic events to resistance to monotherapy and the emergence of adaptive resistance. Others have explored mechanisms of adaptive resistance to tipifarnib [33] and have proposed cellular events including activation of EGFR and FGFR1, increased GTP loading of wild-type NRAS and KRAS [33], de novo *NF1* and *GNAS* mutations [33], downregulation of pathways related to protein localization [65] and increased transcriptional and translational expression of insulin-like growth factor-binding protein 7 (IGFBP7), midkine (MDK), and beta-2-microglobulin (B2M) [56]. It has also been hypothesized that co‐occurring *PIK3CA* mutations may activate a parallel biochemical pathway that could limit the efficacy of inhibiting MEK/ERK signaling [66]. The xenograft SJRHB000026 [67] (HRAS G13R, *PIK3CA* missense mutation) had less profound tumor growth inhibition compared to other HRAS-mutant xenografts, which may be at least in part due to the concomitant *PIK3CA* mutation and the emergence of early adaptive or acquired resistance. Potential combination therapies will need to be explored and may take into consideration factors such as co-occurring mutations, adaptively upregulated pathways, and genomic bypass pathways, as well as agents that can be safely combined with non-overlapping toxicities.

The present study represents the first comprehensive preclinical evaluation of the efficacy of FTI in preclinical models of rhabdomyosarcoma, and in pediatric solid tumors models altogether. Tipifarnib effectively inhibited HRAS processing and demonstrated potent antitumor activity in cell lines and xenografts in which HRAS is the driver oncogene, independent of the specific mutant HRAS allele. Our encouraging preliminary results support ongoing efforts to develop a genomically-driven and histology-agnostic basket trial for pediatric patients with activating mutations in HRAS, a trial that has now begun recruitment within the infrastructure of the Children’s Oncology Group Pediatric MATCH program.

## Materials and methods

### Cell lines, antibodies, and reagents

Human RMS cell lines SMS-CTR and RD were obtained from American Type Culture Collection (ATCC). RH36 was provided by Dr. David Loeb (The Children’s Hospital at Montefiore, Bronx, New York), and CCA, RMS-YM, RH18, JR1 cell lines were provided by Dr. Marielle Yohe (National Institutes of Health, Rockville, Maryland). SJRHB000026_X1 (SJRHB26) was provided by Dr. Elizabeth Stewart (St. Jude Children’s Research Hospital, Memphis, Tennessee). Patient-derived RMS cell lines JH-ERMS-1 and JH-ERMS-2 were generated in our laboratory from biospecimens collected during surgical resection from pediatric patients with RMS. Material was collected under an institutional review board (IRB)-approved protocol and all patients provided written informed consent. JH-ERMS-1 was established first as a PDX, and then subsequently cells were cultured in vitro from the tumor derived in the mouse. JH-ERMS-2 was established as an in vitro cell culture without requiring passage through the mouse. SK-ERMS-2B was developed as a patient-derived cell line by Dr. Romel Somwar (Memorial Sloan Kettering Cancer Center, New York City, New York). Cell lines were authenticated using short tandem repeat (STR) analysis to confirm their identity against published STR profiles, where available. STR was used to confirm that patient derived cell lines (JH-ERMS-1 and JH-ERMS-2) were matches to the patient tumor from which they were derived. The STR profiles are provided as Supplemental Data Table 1. RMS cell lines were characterized using Sanger sequencing and next-generation targeted sequencing. *HRAS, NRAS* and *KRAS* mutants were identified by the presence of mutations in codons 12, 13 or 61 and data are summarized in Supplemental Fig. 1. SJRHB000026_X1 and JH-ERMS-1 used as cell lines were tested for the presence of mouse DNA, which was positive in both lines, consistent with their origin as human- murine PDX. The continued expression of mutant HRAS and histologic appearance compatible with rhabdomyosarcoma served as validation of their identity as described.

Wild-type HRAS, HRAS_Q61K, HRAS_Q61L, HRAS_G12V, KRAS_G12V and NRAS_Q61L expression constructs were obtained from Addgene, and pBABE-containing retrovirus was produced to transduce C2C12 cells and generate RAS and RAS-mutant over-expressing stable clones. The base medium for SJRHB000026 and CCA is Dulbecco’s Modified Eagle Medium (DMEM) and for JH-ERMS-1 and JH-ERMS-2 is Dulbecco’s Modified Eagle Medium/Nutrient Mixture F-12 Ham (DMEM:F12). All other cell lines were cultured in Roswell Park Memorial Institute (RPMI) 1640. All growth medium was supplemented with 10% fetal bovine serum (FBS), 2 mM L-glutamine and 1% penicillin G (50 U/ml) and streptomycin sulfate (50 μg/ml). Cell lines were maintained in a humidified 37 °C incubator with 5% (all other cell lines) or 7% CO2 (CCA). All cell lines tested negative for mycoplasma contamination.

Antibodies were purchased from Abcam Inc (Cambridge, MA): Goat Anti-Rabbit IgG H&L (Alexa Fluor 488) (cat # A32731), from Santa Cruz Biotechnology (Dallas, TX) EGFR (cat # sc-373746); from Cell Signaling Technology (CST) (Danvers, MA): MEK 1/2 (cat # 9122 S), pMEK (S217/221) (cat # 86128), ERK 1/2 (cat # 9102), pERK1/2 (T202/Y204) (cat # 4370), pS6 (S235/236) (cat # 4858), S6 (cat # 2317), pan-RAS (cat #8821), vinculin (cat # 12901), actin (cat # 3700), beta-tubulin (cat # 2128), GAPDH (cat # 5174), cleaved PARP (cat # 5625), total PARP (cat # 9532), cleaved caspase 3 (cat # 9664), pAKT (S473) (cat # 9271), AKT (cat #4685), Rac 1/2/3 (cat # 2465), RhoA (cat # 2117); from Proteintech (Rosemont, IL): HRAS (cat #18295-1-AP) and NRAS (cat #10724-1-AP). Antibodies were used for immunoblots at a dilution of 1:1,000. Tipifarnib was provided by Kura Oncology under a JHU institutional-approved Material Transfer Agreement. Drugs for in vitro studies were dissolved in DMSO to yield 10 mmol/L stock solutions, and stored at –80 °C.

### Immunoblotting

Immunoblotting was performed as described previously [68]. Cells were plated at 2 × 10^6^ cells per well in 10 cm plates and incubated with either tipifarnib treatment or DMSO. Cells were harvested by centrifugation, washed with ice-cold phosphate-buffered saline, and lysed in a buffered solution containing phenyl-methane sulfonyl fluoride, sodium orthovanadate. Protein concentration was determined with Pierce BCA Protein Assay Kit (Thermo Fisher Scientific) on a microplate reader (SpectraMax M5). Equal amounts of proteins were resolved on 10% or 12% SDS-polyacrylamide gels and transferred to nitrocellulose membrane (Thermo Fisher Scientific). Membranes were probed with primary antibodies and incubated overnight in 4 °C. Following overnight incubation, membranes were incubated with secondary horseradish peroxidase (HRP)-conjugated antibodies for 1 hour at room temperature. Chemiluminescence with the ECL detection reagents, Immobilon Western chemiluminescent HRP substrate (# WBKLS0500, Millipore) or Pierce ECL western blotting substrate (# 32106, Thermo Fisher Scientific) was determined. The membranes were imaged on the ChemiDoc Touch Imaging System (Bio-Rad). All experiments shown were replicated at least twice.

### Active RAS detection/ immunoprecipitation assay

RAF1-RBD immunoprecipitation was performed as previously described [69]. Cells were seeded in 10 cm dishes. The following day, the 70–80% confluent cells were collected, and GTP-bound RAS was isolated using the active RAS detection kit (# 8821) from Cell Signaling according to manufacturer instructions. All experiments shown were replicated at least twice.

### Subcellular fractionation

Cytosolic and membrane fractions were prepared per the manufacturer instructions (Thermo Scientific, #78840). Protein concentrations were determined using Pierce BCA Protein Assay Kit (Thermo Scientific) and read on a microplate reader (SpectraMax M5). Equal amounts of protein were loaded and immunoblotting was performed as previously described. Densitometric analysis of blots were conducted using Image J. All experiments shown were replicated at least twice.

### Immunofluorescence

RMS cell lines were plated at a density of 1 × 10^5^ cells on glass coverslips in 6-well plates. Cells were incubated in 1000 nM tipifarnib for 24 h prior to washing in PBS and fixing in 4% paraformaldehyde at room temperature for 10 min. Cells were then incubated in blocking buffer (10% goat serum) for 1 hour at room temperature. Cells were incubated overnight at 4^o^C in 1:100 dilutions of each HRAS (Proteintech, Catalog number: 18295-1-AP) and NRAS (Proteintech, Catalog number: 10724-1-AP) antibodies in blocking buffer (1% goat serum). Cells were then incubated in 1:1000 goat anti-mouse or goat anti-rabbit AlexaFluor488 with filamentous actin stain in blocking buffer for 1 h at room temperature in the dark. After staining, slides were counterstained with a 1:10,000 dilution of 50 µg/ml 40,6-diamidino-2-phenylindole (DAPI; Sigma Aldrich; # D9542; 5 mg/mL) for 5 min to visualize the nuclei of all cells. Coverslips were mounted with Prolong Diamond mounting medium reagent. After washing, cells were imaged on a Leica SP8 scanning confocal microscope with a ×63 oil immersion lens. All experiments shown were replicated at least twice.

### Viability assay and IC_50_ calculations

3 × 10^3^ cells/well were plated in 96-well plates and treated with DMSO (control) or a range of doses of tipifarnib. 96 h later, medium was removed and alamarBlue (BioRad cat # BUF012B) cell viability assay reagent was added at 1:10 ratio in culture media. After 4 h incubation, fluorescence was measured using CLARIOstar plate reader as per manufacturer instructions. Percent viability normalized to DMSO control was calculated using GraphPad Prism 8 and half maximal inhibitory concentration (IC_50_) values were determined using a four-parameter fit nonlinear regression analysis. Error bars represent mean of three measurements ± SD of mean. All experiments shown were replicated at least twice.

### IncuCyte ZOOM live cell imaging system

1.5 × 10^3^ to 2.5 × 10^3^ cells/well were plated in 96-wells plates and treated with DMSO (control) or a range of doses of tipifarnib. Real-time evaluation of cell confluence was performed using IncuCyte ZOOM (Essen BioSciences), and images were acquired every 4 h. The percentage of cell confluence was measured and analyzed using the IncuCyte ZOOM software. Error bars represent mean of six measurements ± SEM. All experiments shown were replicated at least twice.

### Colony formation assay

1 × 10^3^ cells/well were plated in 12-well plates and treated with DMSO control or indicated doses of tipifarnib. After approximately two weeks the media was removed, and cells were fixed and stained with 0.1% crystal violet in 4% paraformaldehyde for 20 min. After rinsing and drying, the plates were scanned with Canon LiDE220 scanner. All experiments shown were replicated at least twice.

### Soft-agar colony formation

Soft-agar assay was performed as described previously [68]. Briefly, 100,000 to 150,000 cells growing in log phase were mixed with 1% agar (Gibco) treated with either DMSO or tipifarnib (10, 30, 100 nM), and plated over a bottom layer of 4% agar in 6-well plates. Cells were incubated at 37 °C for 3 weeks. Colonies were stained with 4- nitro blue tetrazolium chloride (Sigma-Aldrich) overnight and imaged via ChemiDoc Touch Imaging System (Bio-Rad). The measurements were based on three replicates for each condition. Images captured within a single experiment were taken at the same magnification and exposure time. All experiments shown were replicated at least twice.

### In vivo mouse studies

NOD scid gamma (NSG, # 005557) female mice were purchased from the Jackson Laboratory. All mouse experiments were approved by the Institutional Animal Care and Use Committee (IACUC) at Johns Hopkins under protocol # MO19M115. Cells at 80% confluency were trypsinized, resuspended in a 1:1 solution of PBS and Matrigel, and injected into the flanks of 8-week-old mice (5–7.5 million cells per flank). Tumor-bearing mice (defined as having palpable tumors) were randomized into groups of 6 animals by an algorithm that distributes animals based on measured volume to achieve the best-case distribution to ensure that each treatment group has similar mean tumor volume and standard deviation. Sample size determination was accounted on the need for statistical power. Vehicle or tipifarnib (20 mg/kg or 80 mg/kg) was administered via oral gavage twice daily based on mean group body weight, with a treatment schedule of 5 days on/2 days off. Investigators were not blinded to the treatment groups. The endpoint of the experiment for efficacy studies was considered 3 weeks on treatment or the longest tumor diameter of 2 cm as per the approved animal protocol, whichever occurred first. Tumors were measured twice weekly by calipering in two dimensions, and tumor volume was calculated by: (L × W^2^)(0.5), where L is the longest diameter and W is the width. Data are shown as mean ± SEM. SMS-CTR, RH36 and SJRHB26 xenograft experiments were replicated twice. All other in vivo experimental cohorts were done once.

### Immunohistochemistry analysis

Immunostaining was performed at the Oncology Tissue Services Core of Johns Hopkins University. Immunolabeling for Ki67 was performed on formalin‐fixed, paraffin embedded sections on a Ventana Discovery Ultra autostainer (Roche Diagnostics). Briefly, following dewaxing and rehydration on board, epitope retrieval was performed using Ventana Ultra CC1 buffer (catalog# 6414575001, Roche Diagnostics) at 96 °C for 48 min. Primary antibody, anti‐ Ki67 (1:200 dilution; catalog# Ab16667, Lot number GR3185488-1, Abcam) was applied at 36 °C for 60 min. Primary antibodies were detected using an anti-rabbit HQ detection system (catalog# 7017936001 and 7017812001, Roche Diagnostics) followed by Chromomap DAB IHC detection kit (catalog # 5266645001, Roche Diagnostics), counterstaining with Mayer’s hematoxylin, dehydration and mounting. Whole slide imaging was performed at the Oncology Tissue Services Core of Johns Hopkins University. Scanning was carried out at ×40 magnification (0.23 microns/pixel) using a Hamamatsu Nanozoomer S210 digital slide scanner (Hamamatsu Photonics, Shizuoka, Japan). WSIs were visualized in Concentriq digital pathology platform (Proscia, Philadelphia, PA).

## Supplementary information

Supplemental Material
