# Correction: Targeting farnesylation as a novel therapeutic approach in HRAS-mutant rhabdomyosarcoma

**DOI:** 10.1038/s41388-022-02342-6

**Published:** 2022-05-09

**Authors:** Patience Odeniyide, Marielle E. Yohe, Kai Pollard, Angelina V. Vaseva, Ana Calizo, Lindy Zhang, Fausto J. Rodriguez, John M. Gross, Amy N. Allen, Xiaolin Wan, Romel Somwar, Karisa C. Schreck, Linda Kessler, Jiawan Wang, Christine A. Pratilas

**Affiliations:** 1 Division of Pediatric Oncology, The Sidney Kimmel Comprehensive Cancer Center, Johns Hopkins University School of Medicine, Baltimore, MD USA; 2 Pediatric Oncology Branch, National Cancer Institute, National Institutes of Health, Bethesda, MD 20892 USA; 3 The Greehey Children’s Cancer Research Institute, The University of Texas Health Science Center at San Antonio, San Antonio, TX USA; 4 Department of Laboratory Medicine and Pathology, David Geffen School of Medicine, UCLA, Los Angeles, CA USA; 5 Department of Pathology, Johns Hopkins University School of Medicine, Baltimore, MD USA; 6 Department of Pathology, Memorial Sloan Kettering Cancer Center, New York, NY USA; 7 Department of Oncology, The Sidney Kimmel Comprehensive Cancer Center, Johns Hopkins University School of Medicine, Baltimore, MD USA; 8 Department of Neurology, Johns Hopkins University School of Medicine, Baltimore, MD USA; 9 Kura Oncology, San Diego, CA USA

**Keywords:** Cancer genomics, Sarcoma

Correction to: *Oncogene* 10.1038/s41388-022-02305-x, published online 22 April 2022

Following the publication of this article a number of copy editing omissions were noted.

These have now been corrected.

