# Supplementary material for: Targeting farnesylation as a novel therapeutic approach in HRAS-mutant rhabdomyosarcoma
Source: Oncogene. 2022 Apr 22;41(21):2973–83. doi: 10.1038/s41388-022-02305-x (PMC9122815; doi:10.1038/s41388-022-02305-x)
Supplement: Supplementary file 1 — Supplemental Material [file 41388_2022_2305_MOESM1_ESM.docx]

Supplementary Information for:

**Targeting farnesylation as a novel therapeutic approach in HRAS-mutant rhabdomyosarcoma.**

Patience Odeniyide^1^, Marielle E. Yohe^2^, Kai Pollard^1^, Angelina V. Vaseva^3^, Ana Calizo^1^, Lindy Zhang^1^, Fausto J. Rodriguez^4,5^, John Gross^5^, Amy N. Allen^1^, Xiaolin Wan^2^, Romel Somwar^6^, Karisa C. Schreck^7,8^, Linda Kessler^9^, Jiawan Wang^1^, Christine A. Pratilas^1,7^

**Supplementary Methods**

**Cell line authentication**

All cell lines were validated for identity using short tandem repeat (STR) analysis and compared to published sequences where available. See Supplementary Table 1.

Novel patient-derived cell lines JH-ERMS-1, JH-ERMS-2, and SK-ERMS-2B were derived from tumor resections from patients with fusion-negative rhabdomyosarcoma, and were authenticated using: STR profiling (to ensure identity against the parent tumor); direct sequence confirmation of HRAS mutation; myogenin and desmin expression via immunohistochemistry; MYOD1 expression via immunoblot (JH-ERMS-1 and JH-ERMS-2); and myogenin and MYOD1 expression via PCR (SK-ERMS-2B). For immunohistochemistry, formalin-fixed cell line plugs were generated from cells that were harvested, pelleted, and then formalin-fixed followed by standard paraffin embedding. Cell line FFPE samples and xenograft tumors were subject to immunohistochemical staining.

**DNA extraction, PCR, and direct Sanger sequencing**

Genomic DNA was extracted from cell lines using a QIAamp DNA Mini Kit (Qiagen Cat No. 51304), and quantified and the quality confirmed via NanoDrop 2000 spectrophotometer. One pair of primers was used to amplify the exon–intron junctions and coding regions of exons 2 and 3 of each of the K-, N-, and HRAS genes (primer sequences in Supplementary Table X). PCR amplification was performed in the presence of 500 ng genomic DNA as the template, 1X High Fidelity PCR Buffer, 2.0 mM MgSO_4_, 0.2 mM dNTP, 0.2 μM primers and Platinum Taq DNA Polymerase High Fidelity (Invitrogen, cat No. 11304-011). The PCR program consisted of 5 minutes at 94°C, followed by 35 cycles of 30 seconds at 94°C, 1 min at 58°C, and 30 seconds at 72 °C, and a final extension step of 7 minutes at 72°C. The PCR products were semi-quantified on a 2.5% agarose gel for purity, in 1X Tris Acetate-EDTA buffer and visualized by staining with ethidium bromide. These PCR products were cleaned up using an E.Z.N.A^®^ Cycle Pure Kit (Omega Biotek, cat No. D6492-02), and then subjected to direct Sanger sequencing. Chromatograms were visualized using DNA baser assembler v5.15.0.

Primer sequences:

HRAS exon 2, forward, 5'-AGGAGACCCTGTAGGAGGA-3'

HRAS exon 2, reverse, 5'-CTATCCTGGCTGTGTCCTG-3'

HRAS exon 3, forward, 5'-AGAGGCTGGCTGTGTGAAC-3'

HRAS exon 3, reverse, 5'-GCAGCGGCATCCAGGACAT-3

**RT-PCR**

RNA was extracted using a Qiagen RNA mini kit and cDNAs were synthesized using SuperScript IV VILO (ThermoFisher) according to the manufacturers’ instructions. *MYOD1*, *myogenin* and *GAPDH* were detected by RT-PCR.

Primer sequences:

MYOD1, forward, 5’-ATGGAGCTACTGTCGCCACCGCT-3’

MYOD1, reverse, 5’-TCAGAGCACCTGGTATATCGGGT-3’

Myogenin, forward, 5’-ATGGAGCTGTATGAGACATCCCC-3’

Myogenin, reverse, 5’-TCAGTTGGGCATGGTTTCATCT-3’

GAPDH, forward, 5’- GGC GCT GAG TAC GTC GTG GAG TCC-3’

GAPDH, reverse, 5’- AAA GTT GTC ATG GAT GAC CTT GG-3’

**Apoptosis assay**

Human Apoptosis Antibody Array was purchased from Abcam (Cambridge, MA, USA) (Cat. Ab134001) for detecting the expression of apoptosis-related proteins according to the manufacturer’s instructions.

***In vivo* mouse studies**

Xenograft models were generated as described in the materials and methods (*In vivo* mouse studies). SMS-CTR xenograft tumors were monitored after the cessation of treatment. SJRHB000026 xenograft tumors were monitored and retreated as indicated.

| **Cell Line** | **Source** | **Report Date** | **AMEL** | **CSF1PO** | **D13S317** | **D16S539** | **D21S11*** | **D5S818** | **D7S820** | **TH01** | **TPOX** | **vWA** | **Notes** |
| --- | --- | --- | --- | --- | --- | --- | --- | --- | --- | --- | --- | --- | --- |
| RH36 | Loeb, JHU | 1/22/16 | X, Y | 11, 12 | 12, 13 | 11, 13 | 31, 32 | 13 | 10, 11 | 9.3 | 8 | 14, 18 | 100% MATCH |
| RD | ATCC | 1/22/16 | X | 10, 11 | 13 | 10, 11 | 28, 29 | 11 | 8, 12 | 9.3 | 9 | 18 | 100% MATCH |
| SK-ERMS-2B | Somwar, MSKCC | 1/22/16 | X | 9, 12 | 11, 12 | 10, 12 | 27, 30 | 8, 13 | 10, 11 | 6, 8 | 11 | 18 | Newly established (MSKCC) |
| SMS-CTR | ATCC | 1/22/16 | X, Y | 12 | 11 | 11 | 29, 31.2 | 12 | 8, 11 | 6 | 8, 12 | 18, 19 | MATCH (except source D16S539 is 10, 11) |
| SJRHB26 | Stewart, St. Jude | 4/22/19 | X | 12 | 12 | 9,11 |  | 12 | 8, 9 | 6 | 8,12 | 15,18 | 100% MATCH |
| JR1 | Yohe, NIH | 1/28/19 | X | 12 | 13 | 12,13 | 26,28 | 11,12 | 11 | 9.3 | 8,9 | 15 | 100% MATCH |
| RH18 | Loeb, JHU | 2/18/19 | x | 10 | 12 | 9,12 | 32.2, 33.2 | 12 | 8,10 | 7 | 8,9 | 15,17 | 100% MATCH |
| RMS-YM | Yohe, NIH | 2/18/19 | X, Y | 11 | 10,11 | 11 | 31,32.2 | 12,14 | 10,11 | 9 | 11 | 18 | 100% MATCH |
| CCA | University of Bologna | 1/28/2021 | X, Y | 12, 13 | 13, 14 | 11, 12 | 28, 32.2 | 11, 13 | 10, 12 | 9 | 9, 11 | 15,17 | 100% MATCH |

**Supplementary Table 1.** Short tandem repeats (STR) profiling data of human RMS cell lines.

|  | **Source** | **Report Date** | **AMEL** | **CSF1PO** | **D13S317** | **D16S539** | **D21S11*** | **D5S818** | **D7S820** | **TH01** | **TPOX** | **vWA** |
| --- | --- | --- | --- | --- | --- | --- | --- | --- | --- | --- | --- | --- |
| JH- ERMS-1  Tumor | Pratilas, JHU | 8/14/20 | X | 8, 10 | 11, 13 | 10 | 27, 29 | 10, 13 | 11, 12 | 7 | 11 | 18 |
| JH- ERMS-1  Cell line | Pratilas, JHU | 8/14/20 | X | 8, 10 | 11, 13 | 10 | 27, 29 | 10, 13 | 11, 12 | 7 | 11 | 18 |
| JH- ERMS-1  Xenograft | Pratilas, JHU | 8/14/20 | X | 8, 10 | 11, 13 | 10 | 27, 29 | 10, 13 | 11, 12 | 7 | 11 | 18 |
| JH-ERMS-2  Tumor | Pratilas, JHU | 8/14/20 | X | 10, 12 | 11, 12 | 9 | 33.1, 37 | 13 | 10 | 6, 7 | 9, 10 | 15, 17 |
| JH-ERMS-2  Cell line | Pratilas, JHU | 8/14/20 | X | 10, 12 | 11, 12 | 9 | 33.1, 37 | 13 | 10 | 6 | 9, 10 | 15, 17 |
| JH-ERMS-2  Xenograft | Pratilas, JHU | 8/14/20 | X | 10, 12 | 11, 12 | 9 | 33.1, 37 | 13 | 10 | 6 | 9, 10 | 15, 17 |

**Supplementary Figures**


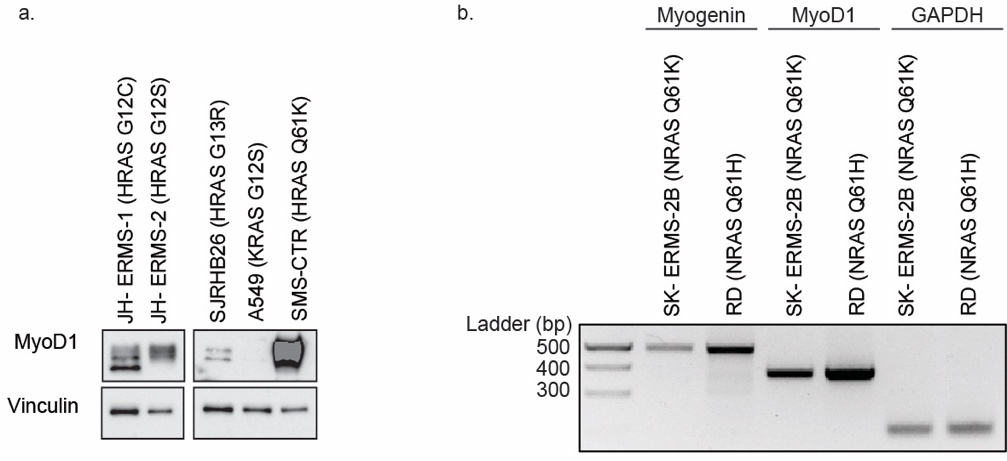


**
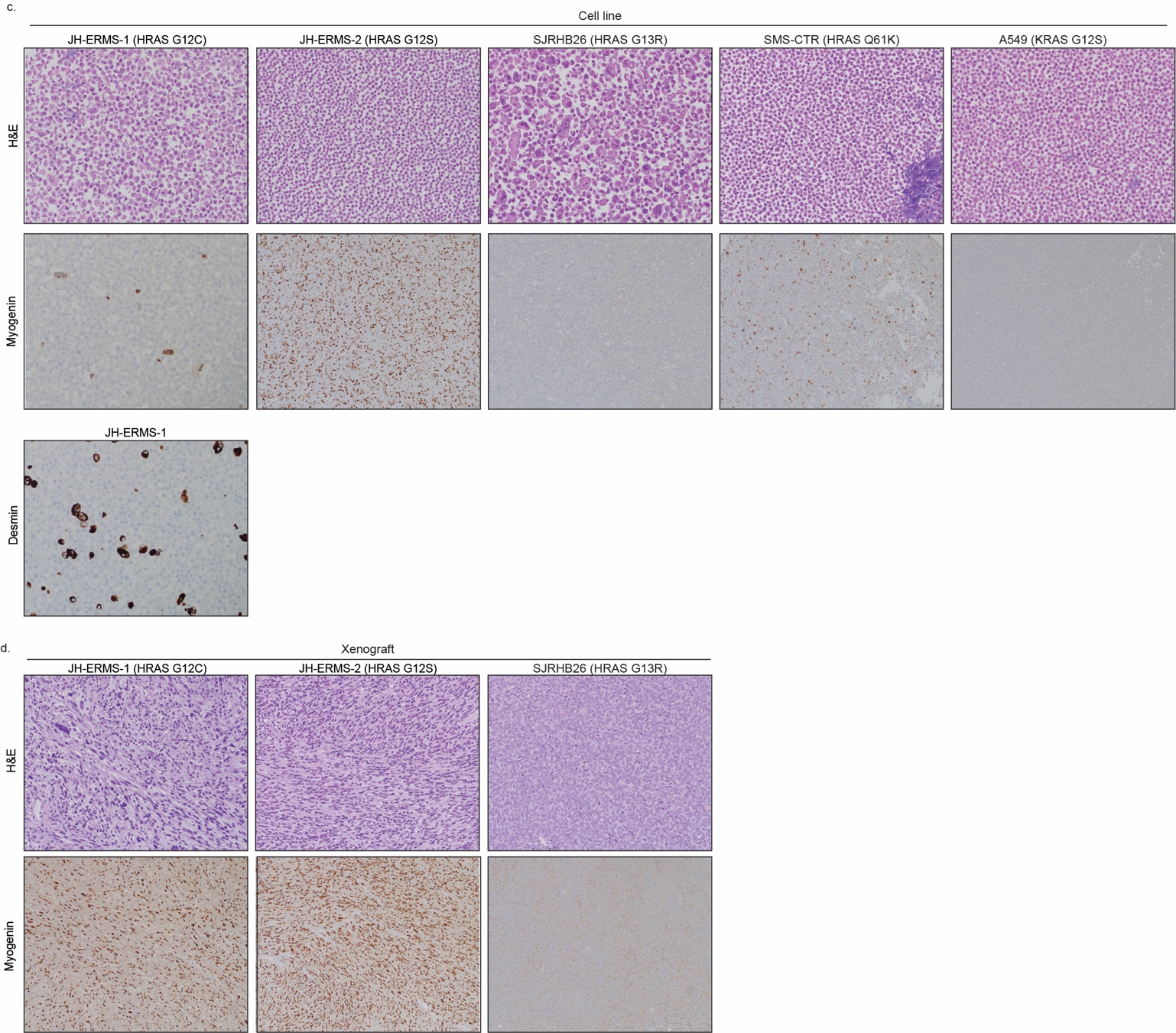
**

 JH-ERMS-1: HRAS G12C, GGC> TGC


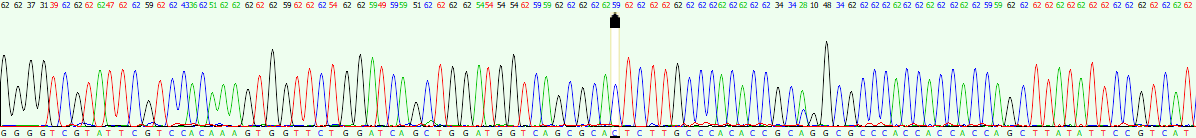


Reverse complemented: CACACCGCAGGCGCC

Sense strand:

GGCGCCTGCGGTGTG

*Mixture of WT G12 and mutant C12

 JH-ERMS-2: HRAS G12S, GGC> AGC


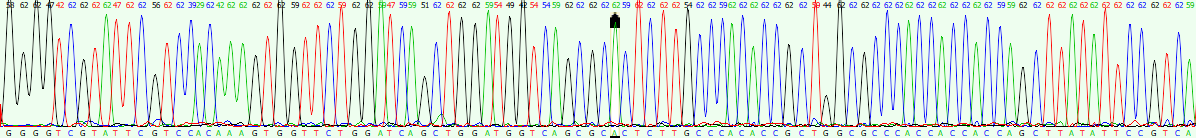


 Reverse complemented: CACACCGCTGGCGCC

Sense strand:

GGCGCCAGCGGTGTG

SJRHB000026 HRAS G13R, GGT> CGT


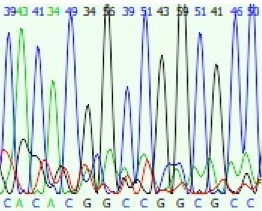


Reverse complemented:

CACACGGCCGGCGCC

Sense strand:

GGCGCCGGC**C**GTGTG

**Sup. Fig 1.**

**Validation of novel RMS cell lines.** **a.** RMS cell lines (mutations as indicated) were subject to immunoblot. MyoD1 and vinculin (loading control) were determined from whole cell lysate (WCL). **b.** RT-PCR analysis of Myogenin, MyoD1 and GAPDH in RMS cell lines.  **c.** Cell line pellets were fixed stained using hematoxylin and eosin (H&E) and subject to immunohistochemistry and stained for myogenin and desmin.  **d.** PDX tumor extracts fixed and stained using hematoxylin and eosin (H&E) and subject to immunohistochemistry and stained for myogenin. **e.** Chromatograms of PCR-amplified products demonstrating mutant HRAS in novel patient derived cell lines.


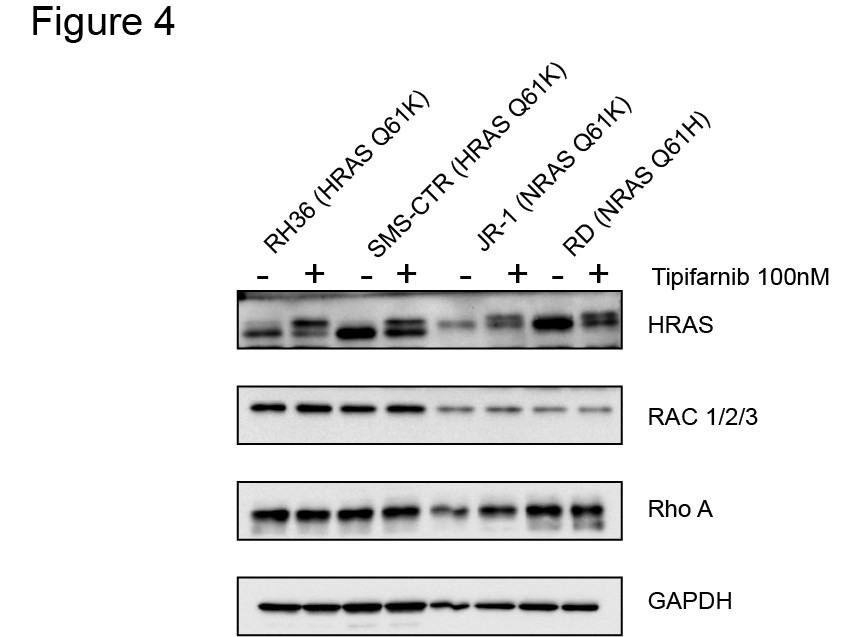


**Sup. Fig 2.**

**Tipifarnib does not effect RAC 1/2/3 and Rho A expression in RMS cell lines.** RMS cell lines (mutations as indicated) were treated with DMSO or 100 nM tipifarnib for 24 hours. HRAS, RAC 1/2/3, Rho A, and GAPDH (loading control) were determined by immunoblot from whole cell lysate.

**
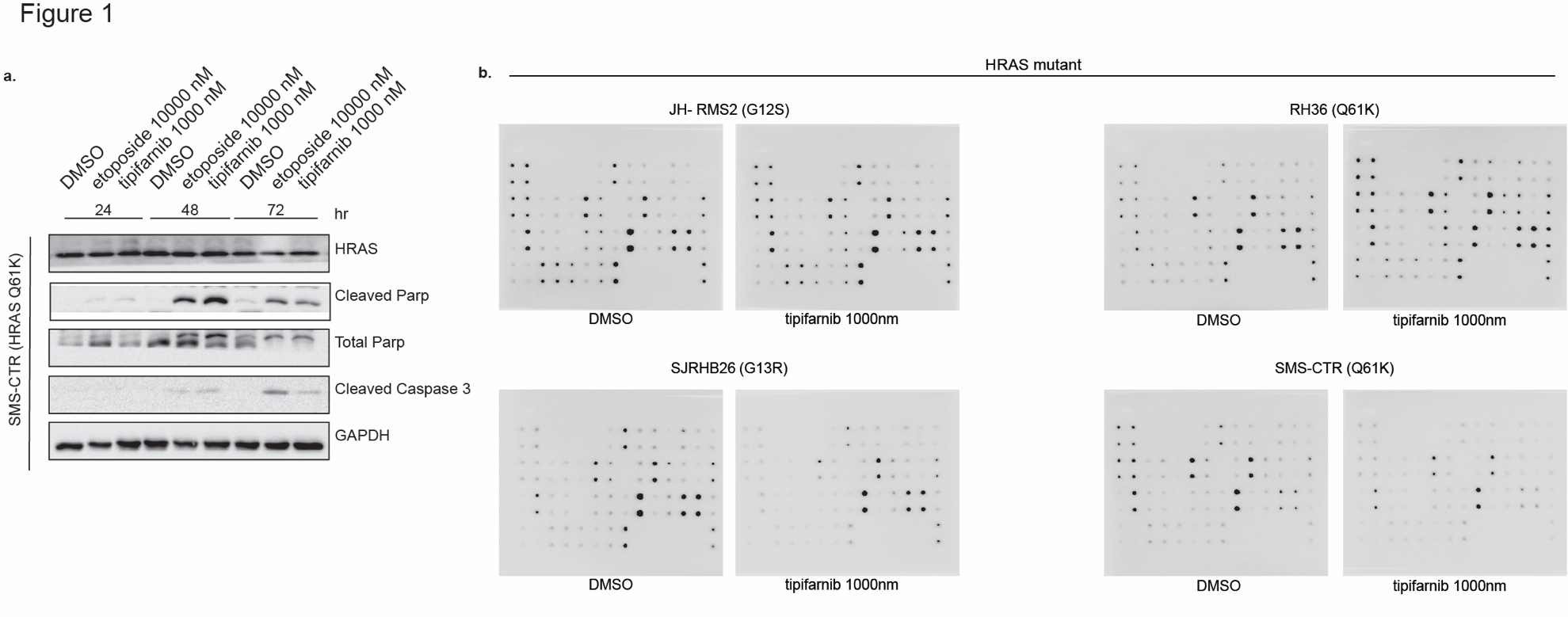
**

**
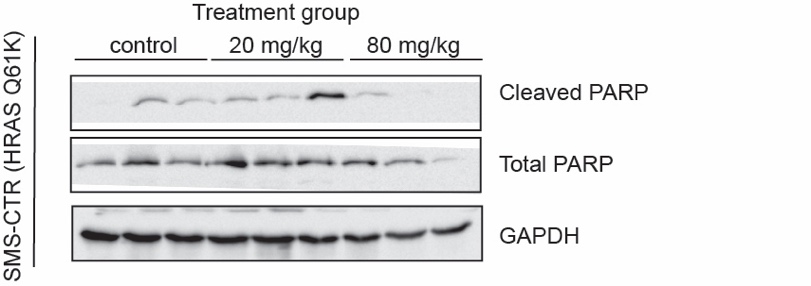
b.**

**
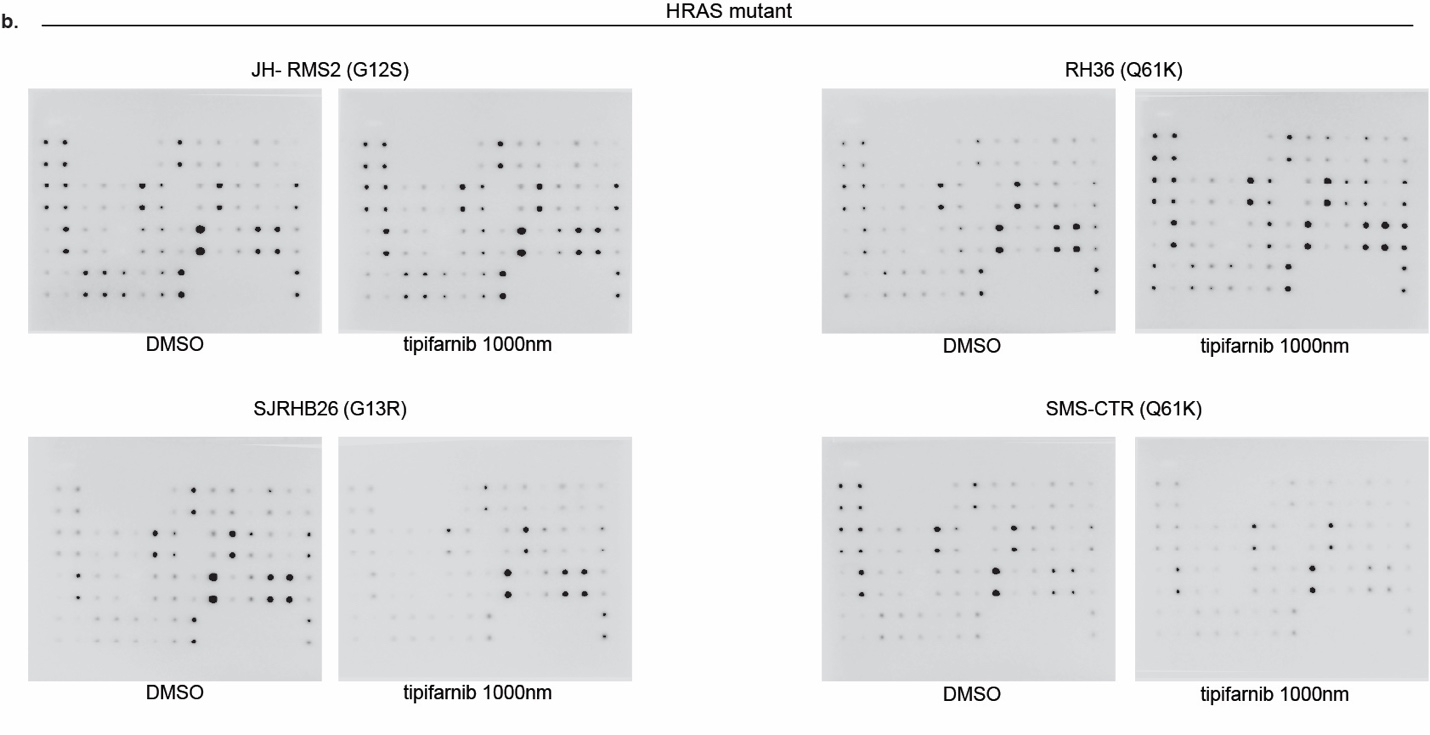
**c.

**Sup. Fig. 3**

SMS-CTR (HRAS Q61K) was treated with DMSO, etoposide (positive control) or tipifarnib. Cleaved and total PARP, cleaved caspase 3, and GAPDH (loading control) were determined by immunoblot from whole cell lysate (WCL). **b.** NSG mice bearing SMS-CTR (HRAS Q61K) xenografts were treated with vehicle, or tipifarnib at 20 or 80 mg/kg twice daily for 10 doses. Cleaved and total PARP and GAPDH (loading control) were determined by immunoblot from whole cell lysate (WCL) **c.** HRAS-mutant cell lines were treated with DMSO or tipifarnib for 48 hours. Expression of apoptosis-related proteins was determined via apoptosis assay.

**
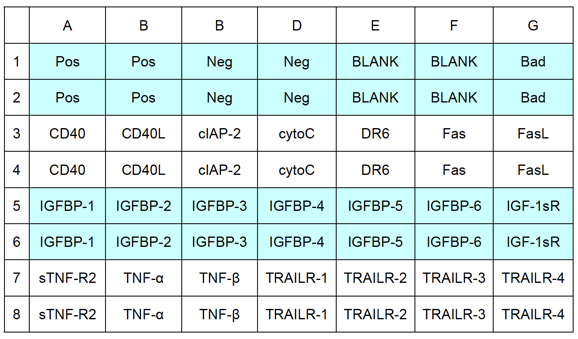

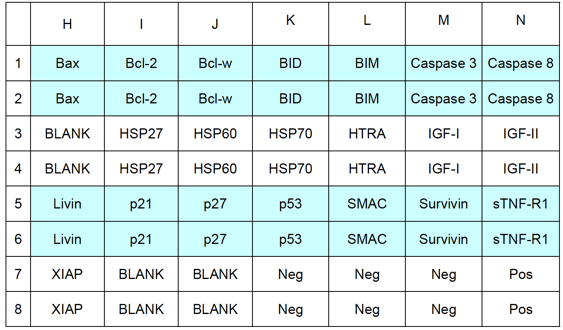
** Apoptosis array key

**
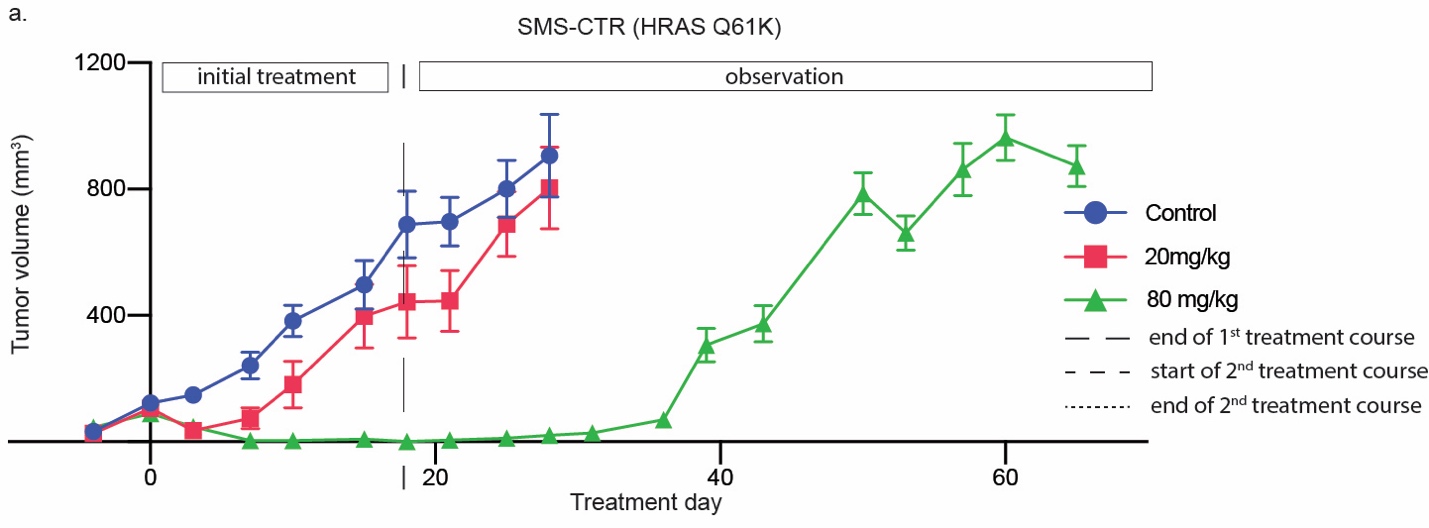
**

**
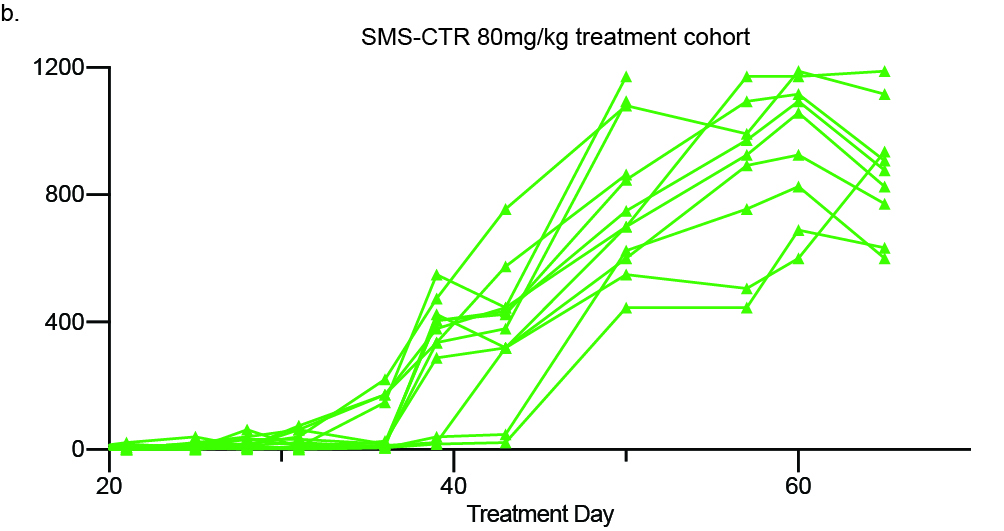
**

**
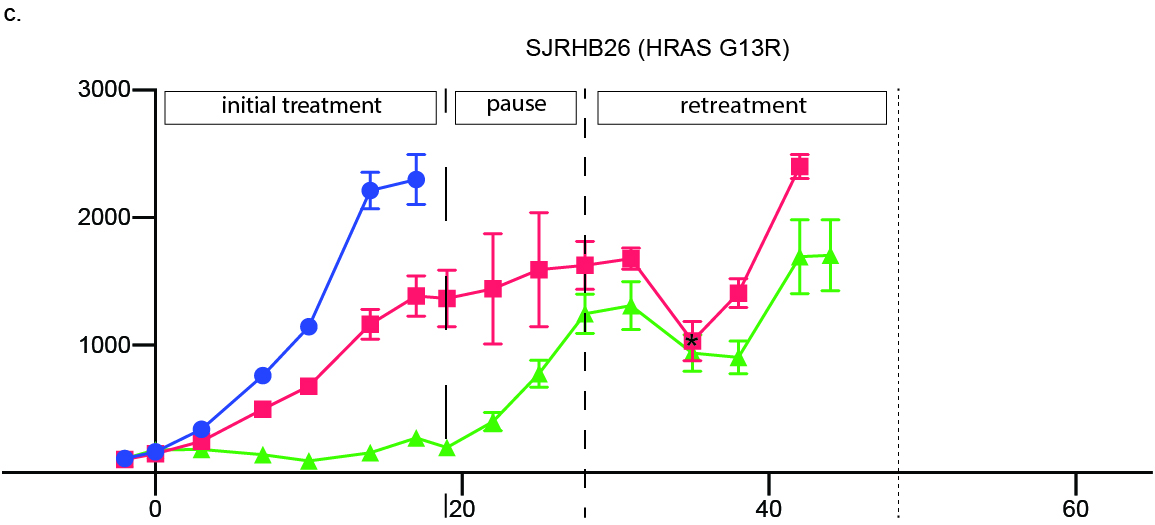
**

**Sup. Fig. 4, related to Fig 4.**

**Tipifarnib preferentially inhibits growth of HRAS mutated RMS xenografts.** (**a**) NSG mice bearing SMS-CTR (HRAS Q61K) xenograft were treated with vehicle, or tipifarnib at 20 or 80 mg/kg twice daily (5 days on/2 days off) for three weeks. Tumor growth was observed following treatment cessation, though day 65. Tumor volumes were calculated twice weekly. The average tumor volume is graphed as a function of days on treatment. Error bars represent mean ± SEM. Tumor volumes from the end of therapy course for tipifarnib 80 mg/kg cohort are illustrated in a spider plot, where each line is the volume from an individual mouse tumor (**b**). SJRHB000026_X1 (HRAS G13R, abbreviated SJRHB26) (**c**) xenografts were treated with vehicle, or tipifarnib at 20 or 80 mg/kg twice daily (5 days on/2 days off). Treatment was paused and tumor growth was observed from days 19 to days 28. Treatment was resumed at previous dosing from days 28 through 44. Tumor volumes were calculated twice weekly. The average tumor volume is graphed as a function of days on treatment. Error bars represent mean ± SEM. * Reflects a drop in tumor volumes in the 20mg/kg treatment group secondary to the death of mice in that cohort rather than a reduction in tumor volumes.


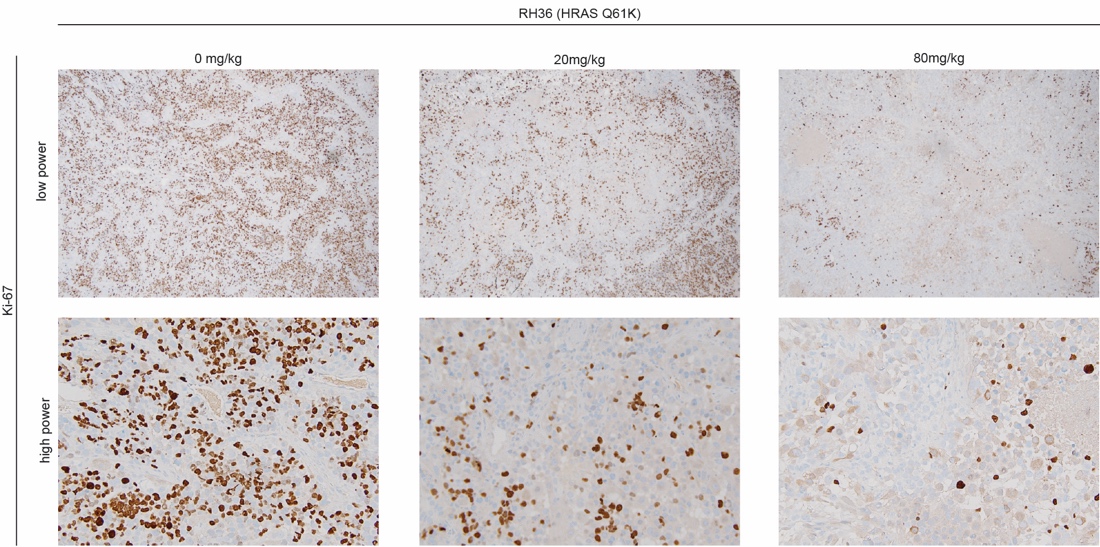


**Sup. Fig. 5, related to Fig 4.**

NSG mice bearing RH36 (HRAS Q61K) xenografts were treated with vehicle, or tipifarnib at 20 or 80 mg/kg twice daily for (5 days on/2 days off) for three weeks. Representative tumor extracts were subject to immunohistochemistry and stained for Ki-67.
